# Intraductal papillary mucinous neoplasm of the biliary tract with cardiac metastasis: A case report

**DOI:** 10.1097/MD.0000000000024310

**Published:** 2021-01-22

**Authors:** Joo Hyung Lee, Hyung Sun Kim, Ji Hyun Park, Joon Seong Park

**Affiliations:** a Pancreatobiliary Cancer Clinic, Department of Surgery; b Department of Pathology, Gangnam Severance Hospital, Yonsei University College of Medicine, Seoul, South Korea.

**Keywords:** cardiac metastasis, intraductal papillary mucinous neoplasm

## Abstract

**Introduction:**

Intraductal papillary mucinous neoplasm of the biliary tract (IPNB) is a rare, low-grade neoplasm limited to the bile duct mucosa. The malignant transformation rate is low, and there have been limited reports of metastasis to other organs. Herein, we presented a rare case of a patient who was diagnosed with IPNB concurrent with invasive adenocarcinoma after surgery and was diagnosed with cardiac metastasis 6 months later.

**Patient concerns:**

A 61-year-old male patient presented with abdominal pain to a local clinic. He was diagnosed with intrahepatic cholangiocarcinoma with pancreatitis and transferred to our hospital.

**Diagnosis:**

Diagnostic testing (magnetic resonance imaging, endoscopic retrograde cholangiopancreatography, positron emission tomography-computed tomography) revealed a papillary neoplasm and invasive adenocarcinoma with papillary neoplasm in the periampullary lesion.

**Interventions:**

Pancreaticoduodenectomy, right hemihepatectomy, and left lateral sectionectomy of the liver were performed. After surgery, we planned gemcitabine-based adjuvant chemotherapy.

**Outcomes:**

Upon completion of the sixth gemcitabine chemotherapy cycle, a hyperechoic, oval-shaped mass (1.3 × 2.6 cm) was found on the outer wall of the right atrium. Resection of a cardiac tumor in the right atrium and patch repair were performed.

**Conclusion:**

To our knowledge, no other case of cardiac metastasis found during observation after surgery for an IPNB has been described. IPNBs are known to be less aggressive and to have a lower metastasis rate than intraductal papillary mucinous neoplasms; therefore, the number of case reports describing metastasis after surgery is relatively low. Our case suggests that close observation is necessary in patients diagnosed with an IPNB.

## 1 Introduction

Intraductal papillary mucinous neoplasms of the biliary tract (IPNBs) are rare, low-grade neoplasms arising from the bile duct.^[1–4]^ They are commonly found in Far East Asia. IPNBs can occur anywhere in the extrahepatic or intrahepatic bile ducts.^[5]^ According to other published papers, the most probable location for an IPNB is the intrahepatic area or the hepatic hilum.^[6–8]^ Like intraductal papillary mucinous neoplasms (IPMNs), IPNBs secrete mucin and induce biliary obstruction and dilation. IPNBs have recently been considered as an important precursor lesion of bile duct cancer.^[9]^ The rate of distant metastasis from IPNBs is yet to be identified. According to some studies, the metastatic rate was higher for intrahepatic IPNBs than for extrahepatic IPNBs (44.5% vs 66.6%).^[10]^ The most prevalent recurrence sites were the peritoneum and retroperitoneal lymph nodes. Kim et al reported that 12 out of 33 patients showed recurrence; the extrahepatic metastasis sites were as follows: lung (2 cases), stomach (1 case), abdominal wall (1 case), and peritoneum (2 cases).^[11]^

Cardiac metastasis is rare and not well known. Several reports have shown a cardiac metastasis rate of 2.3% to 18.3%, and the metastatic sites involved include the pericardium, epicardium, and myocardium.^[12,13]^ The pericardium was the most common metastatic site. Cardiac metastasis occurred more often in cases of melanoma, primary mediastinal tumors, lung cancer, breast cancer, and hematologic malignancies such as non-Hodgkin lymphoma.^[12,14–16]^

Herein, we presented a rare case of a patient who was diagnosed with IPNB with concurrent invasive adenocarcinoma after surgery and subsequently developed cardiac metastasis, confirmed as metastatic adenocarcinoma.

## 2 Case report

**Figure 1:**
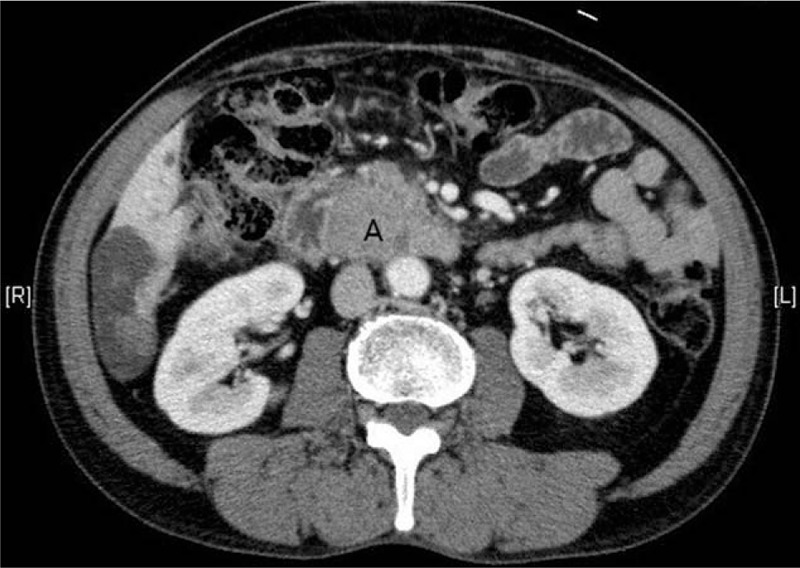
Abdominopelvic computed tomography showing a 4 × 4.5-cm-sized solid enhancing lesion in the periampullary region.

**Figure 2:**
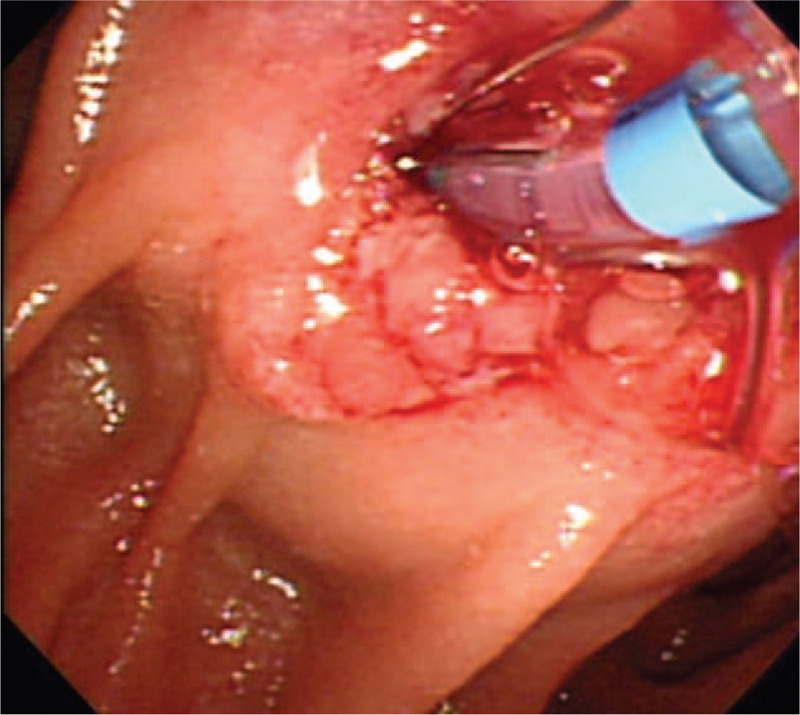
Endoscopic retrograde cholangiopancreatography showing a periampullary fungating mass.

**Figure 3:**
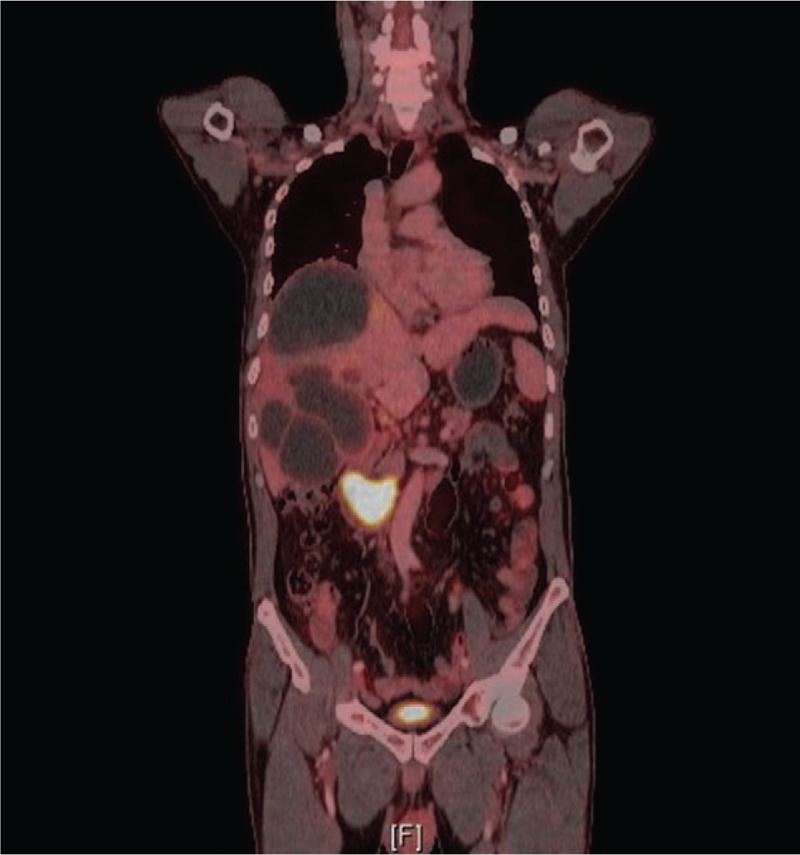
Positron emission tomography showing a small hypermetabolic lesion in the second portion of the duodenum, possibly in the ampulla of Vater area.

A 61-year-old male patient presented with a 2-week history of abdominal pain and a 1-day history of dyspepsia. At a local clinic, the patient was diagnosed with intrahepatic cholangiocarcinoma with pancreatitis and transferred to our hospital. He had no pertinent medical or family history. Computed tomography (CT) at the previous hospital revealed biliary cyst adenocarcinoma with peripancreatic metastases and a malignant IPMN in the periampullary portion (Fig. 1). We performed magnetic resonance imaging, which revealed multiple multifocal, dilated, cystic lesions in the liver (maximum transverse diameter: 9.5 cm) with multifocal internal solid lesions mainly in the peripheral portion of the dilated duct. In addition, a solid enhancing lesion sized approximately 4 × 4.5 cm was observed. Based on this, we suspected a multifocal malignant intraductal papillary tumor of the bile duct with metastatic lymph nodes (2.4 cm) or biliary cystadenocarcinoma with peripancreatic lymph node metastases. Mild extrahepatic bile duct dilatation (approximately 2 cm) was observed and considered to be a probable secondary change due to external compression of the distal common bile duct by the peripancreatic metastasis. The levels of tumor markers were as follows: alpha-fetoprotein 1.5 ng/mL (normal range 1.1–5.0 ng/mL), carcinoembryonic antigen 1.9 ng/mL (normal range 0.2–5.0 ng/mL), and cancer antigen 19-9 159.4 ng/mL (normal range 0.8–24.0 ng/mL). A liver biopsy performed at the previous hospital revealed glandular proliferation cells showing a tubulopapillary structure. We performed a repeat biopsy via endoscopic retrograde cholangiopancreatography. A fistula was observed in the periampullary fungating mass and the second portion of the duodenum (Fig. 2). Biopsy of the periampullary fungating mass showed a papillary neoplasm and an invasive adenocarcinoma. Positron emission tomography-computed tomography revealed biliary cystadenocarcinomas in the liver, a malignancy in the ampulla of Vater area, and a 3.7-cm sized well-defined hypermetabolic mass in the pancreaticoduodenal groove (Fig. 3).

After diagnosis, pancreaticoduodenectomy, right hemihepatectomy, and left lateral sectionectomy of the liver were performed. Intraoperatively, there were no specific findings such as adhesion, and after confirming that there was no tumor in the bile duct and no positive pancreatic duct resection margins, anastomosis was performed with the duct-to-mucosa method using a short stent.

**Figure 4:**
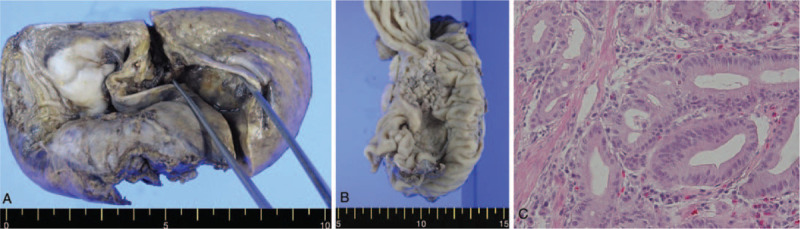
Specimen analysis. A: Estimation of cystic dilatation of the intrahepatic duct. B: The mass in the periampullary area appears to extend to the ampulla by forming the intraductal papillary neoplasm located in the distal common bile duct. C: Pathologic examination of the specimen: Tall columnar cells associated with an intraductal papillary neoplasm (hematoxylin-eosin, × 200).

After surgery, the patient was discharged without complications. Pathologic examination revealed an intraductal papillary neoplasm (biliary papillomatosis) with high-grade intraepithelial neoplasia in the right lobe; cystic dilatation with inflammation, fibrinoid necrosis, and hemorrhage in the left lobe; and an intraductal papillary neoplasm with an associated poorly differentiated invasive adenocarcinoma in the periampullary area. The mass in the periampullary area appeared to extend to the ampulla by the intraductal papillary neoplasm (biliary papillomatosis) located in the distal common bile duct rather than to the primary ampulla neoplasm. The mass had the same shape as the liver tumor, and the 2 lesions may have been connected along the bile duct (Fig. 4A–C). Immunohistochemical staining showed positivity for MUC-1, MUC-2, MUC-5, MUC-6, and glucose transporter (Glut)-1 and negativity for CDX-2.

**Figure 5:**
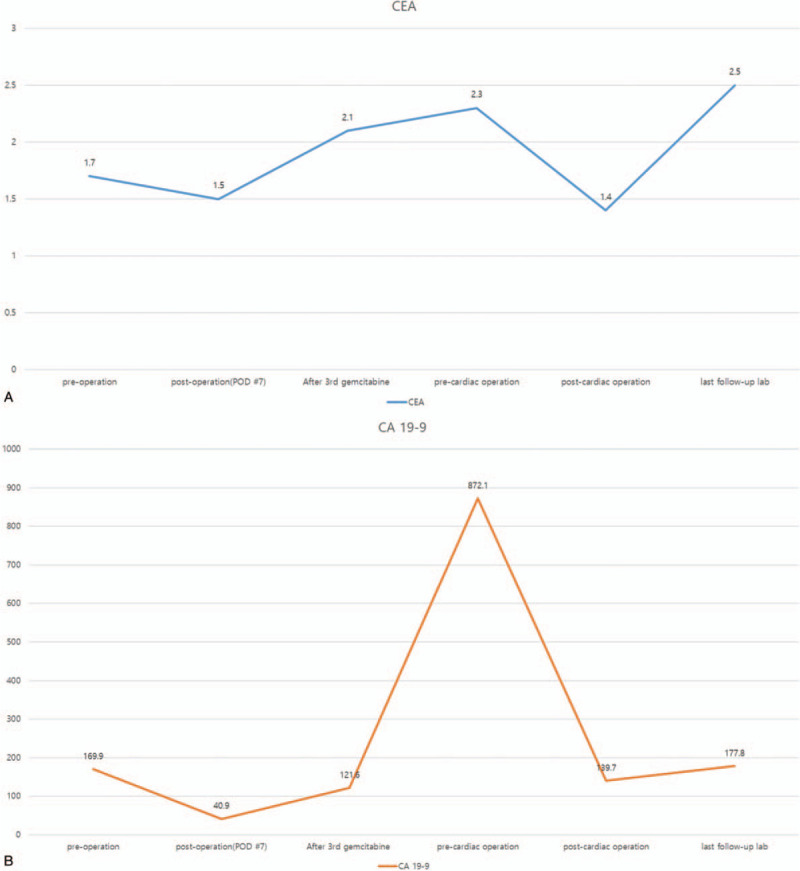
A, B: Graphs depicting a gradual increase in the levels of carcinoembryonic antigen (CEA) and cancer antigen (CA).

Therefore, we diagnosed an intraductal papillary neoplasm (biliary papillomatosis) with high-grade intraepithelial neoplasia (Stage IV based on the cancer staging manual of the American Joint Committee on Cancer [AJCC], 7th edition) and planned gemcitabine-based adjuvant chemotherapy (1700 mg of gemcitabine [1000 mg/m^2^ × body surface area (BSA), 1.7 m^2^ of BSA] once a week for 3 weeks with 1 week of rest). The patient had no subsequent complications and showed stable disease on follow-up CT performed after the third cycle of gemcitabine chemotherapy, based on the Response Evaluation Criteria in Solid Tumors. However, the levels of tumor markers (carcinoembryonic antigen and cancer antigen 19-9) increased (Fig. 5A-B). Regardless, chemotherapy was continued as originally planned because there were no signs of recurrence or other specific findings on CT.

**Figure 6:**
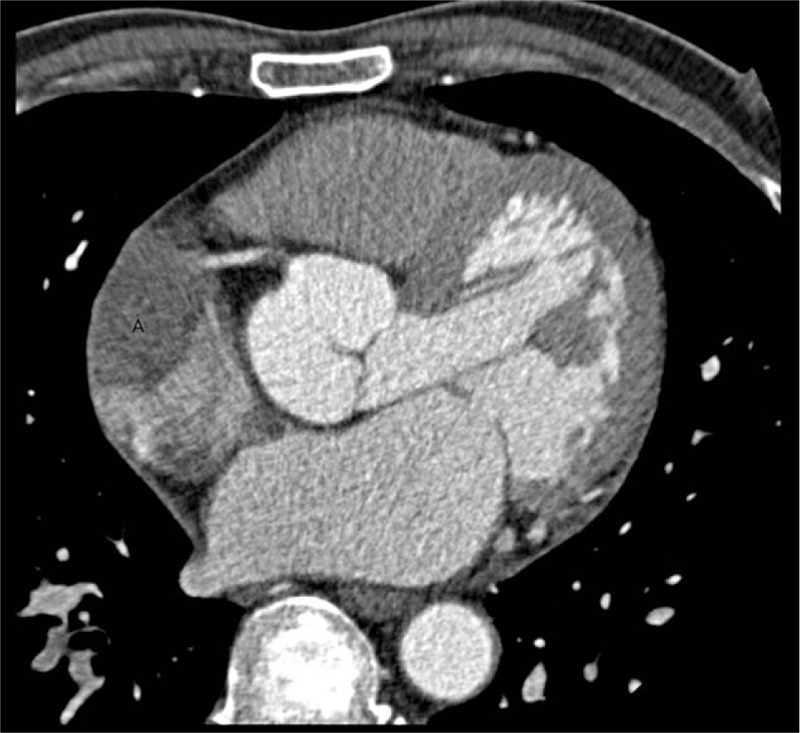
Computed tomography showing a 3.5-cm filling defect at the right atrium of the heart.

**Figure 7:**
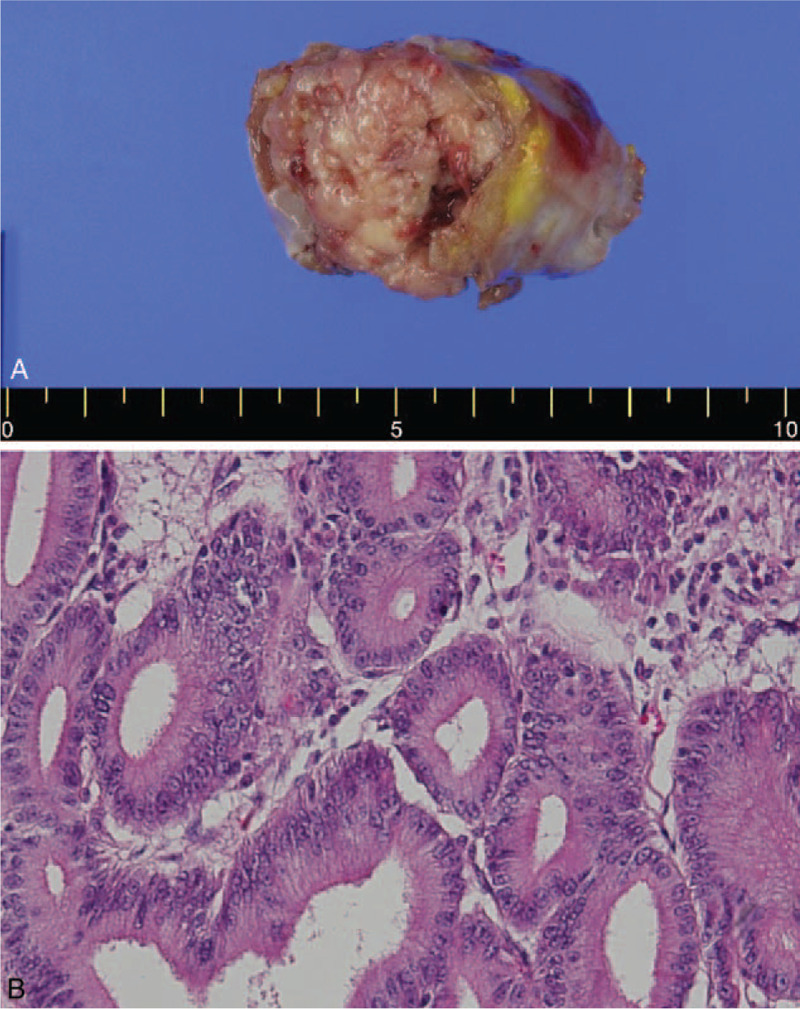
Specimen analysis and pathology. A: The specimen was 5 × 4 × 3 cm in size, and the weight was 20.5 g. A fungating multilobulated soft mass (about 3.5 cm) is seen in the internal cardiac chamber. B: Pathologic examination of the specimen: On multiple sections, multilobulated, yellowish, solid masses are seen. The cardiac mass is an adenocarcinoma composed of tall cells infiltrating cardiac muscle bundles (hematoxylin-eosin, × 200).

After completion of the sixth cycle of gemcitabine chemotherapy, the patient noted constant bruising in his leg and complained of pain. The symptoms did not improve, and therefore, CT was performed, which confirmed a 3.5-cm filling defect in the right atrium of the heart (Fig. 6). Transthoracic echocardiography revealed a hyperechoic oval-shaped mass (1.3 × 2.6 cm) on the outer wall of the right atrium, suggesting a metastatic lesion. Therefore, we proceeded with surgery. Resection of the cardiac tumor and patch repair were performed successfully. Gross examination revealed a fungating multilobulated soft mass (about 3.5 cm in size) in the internal cardiac chamber. Histopathologic examination confirmed metastatic adenocarcinoma from the pancreatobiliary tract (Fig. 7A-B).

**Figure 8:**
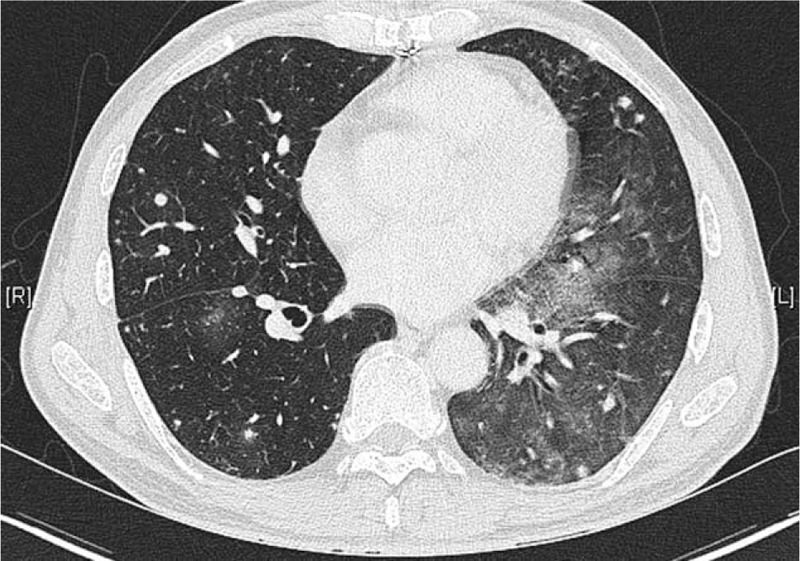
Chest computed tomography showing extensive metastatic lesions in the bilateral lung fields.

Subsequently, palliative 5-fluorouracil chemotherapy was planned. The patient was briefly lost to follow-up for 6 months and did not receive chemotherapy. After 6 months, he was readmitted for hemoptysis symptoms. CT showed extensive metastatic lesions in both lung fields. Bronchoscopy was performed, and active bleeding was confirmed in the anterior and apico-posterior segments of the left upper lobe. Aspiration of blood toward the basal segment was also identified (Fig. 8). After the embolization of both bronchial arteries, the patient no longer complained of symptoms. Subsequently, the patient was admitted to another hospital for palliative care.

## 3 Discussion

Monma et al reported a patient who required surgery after an IPMN metastasized to the right atrium.^[17]^ As mentioned in the introduction, a cardiac tumor is far more likely to be a metastatic tumor than a primary tumor. Cardiac metastases are seen in 36% to 39% of lung cancers, 10% to 12% of breast cancers, and 10% to 21% of hematologic cancers.^[12,18]^ It is known that the mechanism of metastasis differs depending on the transition site. For example, direct invasion occurs in the case of pericardial metastasis, whereas metastasis to the right atrium or right ventricle occur via hematogenous or lymphatic spread.^[19]^ In general, it is known that the prognosis is poor in cases of metastasis from various primary cancers to the heart, and the rate of detection at autopsy was confirmed to be about 7.1%.^[12,20]^

Cardiac metastases from biliary cancers are not well known. A paper published in 2013 reported that there were only 7 known cases of cardiac metastases from gallbladder cancer.^[21]^ As the overall survival rate of cancer patients increases with improvements in treatment, the rate of cardiac metastasis is also increasing. However, the rate of metastasis to the heart is lower than that of metastasis to other organs. To our knowledge, no case has been published describing cardiac metastasis after the resection of an IPNB. Furthermore, the rate of metastasis from an IPNB to organs other than the heart has been reported to be low.^[22]^ In our case, the identified periampullary lesion was accompanied by invasive adenocarcinoma.

In the current case, metastasis occurred to the right atrium and was thought to be caused via a hematogenous or lymphangitic mechanism. Since pulmonary metastases are subsequently observed, we suggest that close clinical follow-up of cases involving IPNB accompanied by invasive carcinoma is needed. Based on our experience and what is already known, it is necessary to reconsider the aggressiveness of IPNB and to confirm the possibility that even IPNB may exhibit more aggressive characteristics in cases involving invasive components.

One limitation of our case report is that the patient was lost to follow-up for 6 months before the appointment during which he was found to have extensive metastatic lesions. Active surveillance is necessary in patients diagnosed with an IPNB due to the potential of metastasis to organs less commonly involved in metastasis from IPNBs.

## Acknowledgments

We would like to thank Editage (www.editage.co.kr) for English language editing.

## Author contributions

**Conceptualization:** Hyung Sun Kim.

**Resources:** Ji Hyun Park.

**Supervision:** Joon Seong Park.

**Writing – original draft:** Joo Hyung Lee.

**Writing – review & editing:** Hyung Sun Kim, Joon Seong Park.
